# The patient’s experience of primary ciliary dyskinesia: a systematic review

**DOI:** 10.1007/s11136-017-1564-y

**Published:** 2017-03-30

**Authors:** Laura Behan, Bruna Rubbo, Jane S. Lucas, Audrey Dunn Galvin

**Affiliations:** 1 Primary Ciliary Dyskinesia Centre, University Hospital Southampton NHS Foundation Trust, Southampton, UK; 2 NIHR Southampton Respiratory Biomedical Research Unit, University of Southampton Faculty of Medicine and University Hospital Southampton NHS Foundation Trust, Southampton, UK; 3 School of Applied Psychology, University College Cork, Cork, Ireland

**Keywords:** Primary ciliary dyskinesia, Patient perspective, Health-related quality of life, Patient experience

## Abstract

**Background:**

Primary ciliary dyskinesia (PCD) is a rare genetic disorder characterised by progressive sinopulmonary disease, with symptoms starting soon after birth. The aim of this study is to critically review, analyse, and synthesise the literature in order to understand the experiences of patients with primary ciliary dyskinesia (PCD) and the impact on health-related quality of life.

**Method:**

MEDLINE, EBSCO, Cumulative Index to Nursing and Allied Health Literature (CINAHL), PsycINFO and EMBASE were searched according to the inclusion criteria. A qualitative analysis of 14 studies was conducted.

**Results:**

Fourteen studies were included in the review, five with qualitative methodologies. Studies originated from the UK, USA, Italy, Denmark and Belgium, one study included a survey distributed internationally. Significant relationships were found between age and worsening of respiratory symptoms, physical, and mental domains of health-related quality of life, with a greater decline compared with reference populations. Variations between the UK and Italy were found for health-related quality of life and its correlation with time since diagnosis. PCD was found to have a physical impact in all age groups: patients found it difficult to keep up with others, and found energy levels were easily depleted compared to family or peers. In terms of social impact, symptoms lead to embarrassment and a sense of isolation, with patients concealing symptoms and/or their diagnosis. In turn, isolation was also linked with the lack of public and medical knowledge. In relation to emotional impact, anxiety was reported in a number of qualitative studies; patients were anxious about getting sick or when thinking about their future health. The burden of treatment and factors influencing adherence were also discussed in depth.

**Conclusion:**

Health-related quality of life decreases with age in patients with PCD. For all age groups, PCD was found to greatly impact physical, emotional, social functioning, and treatment burden. More research is needed on the psychosocial impact of the illness, disease burden and its effect on quality of life.

## Background

Primary ciliary dyskinesia (PCD) is a rare, inherited lung disease affecting cilia motility such that mucociliary clearance is impaired. Individuals with PCD often present with unexplained neonatal symptoms such as neonatal cough, rhinitis transient tachypnoea, and pneumonia, often requiring respiratory support [1–3]. Patients continue to have persistent sinopulmonary symptoms in infancy. Chronic and progressive chest symptoms persist throughout life and include daily wet cough and recurrent chest infections which almost consistently lead to bronchiectasis [4, 5]. By adulthood, bronchiectasis is present and some patients develop respiratory failure [2]. Upper airway symptoms include rhinosinusitis and recurrent serous otitis media with hearing impairment [6]. Situs inversus is found in approximately 50% of cases and situs ambiguous is seen in approximately 10% of cases [6, 7].

Assessment on the prevalence, burden of disease, and prognosis of PCD patients is difficult to determine due to a lack of representative international data. Reported prevalence varies from 1:2000 to 1:40,000; this could reflect true variability or could be a result of poor access to diagnostic facilities in some areas and countries [8–10]. A European Respiratory Society (ERS) Task Force survey of 26 European countries found that PCD is both under-diagnosed and diagnosed late [8].

As in most orphan diseases, research has focused on describing the pathophysiological mechanisms of the illness and improving diagnostics. Few studies have examined the psychosocial impact of the illness, disease burden, and its effect on health-related quality of life. This was highlighted by McManus back in 2003 [11], where a systematic search found no studies reporting from the patient perspective on impact to daily functioning, or on mental health and well-being.

The overall aim of this study was to synthesise the results from both qualitative and quantitative studies which examine the psychosocial impact of PCD. Through this synthesis, we evaluated qualitative studies documenting the experiences and views of PCD patients, the impact of the condition on their daily lives, in addition to health-related quality of life and any influencing factors. We included all age groups (adults, children, adolescents) and parents of PCD children. The qualitative studies allowed us to identify the most salient themes among age groups through interviews and focus groups analysis. The quantitative studies allowed us to compare patient-reported outcome measures (PROMs) and factors influencing variability. Finally, through this synthesis, we assessed the quality of the studies and made recommendations on future research needs.

## Method

### Search strategy

The systematic review was conducted using the Preferred Reporting Items for Systematic Review and Meta-Analyses Approach (PRISMA) [12]. The following electronic databases searched for papers published in the English language from inception until September 2015: MEDLINE–EBSCO, Cumulative Index to Nursing and Allied Health Literature (CINAHL), PsycINFO and EMBASE. Keywords and subject headings/MeSH terms searched in titles and abstracts using various combinations included: “ciliary dyskinesia, primary”, “ciliary motility disorder”, “Kartagener’s syndrome”, “primary ciliary dyskinesia”, “perspective”, “perception”, “knowledge”, “opinion”, “psychological”, “experience”, “attitude”, “impact”, “view”, “idea”, “quality of life”, “QOL”, “HRQL”, “patient report”, “belief”, and “awareness”.

### Inclusion and exclusion criteria

Inclusion criteria were primary studies that reported on experiences and perspective of PCD patients of all age groups or where patients completed PROMSs as primary or secondary outcomes. Quantitative, qualitative, and mixed methodologies were considered equally. PROMs were operationalised as generic health-related quality of life questionnaires, e.g. Short Form-36 (SF-36), and disease-specific health-related quality of life questionnaires, e.g. St. George’s Respiratory Questionnaire (SGRQ) and Leicester Cough Questionnaire (LCQ). Measures of psychological distress, e.g. Child Behaviour Checklist questionnaire and Parenting Stress Index–Short Form, were also included. Qualitative studies and mixed methods studies with a significant qualitative component were considered for inclusion if the number of participants was greater than one, and if sufficient methodological details and data were provided. Non-primary research articles (letters, commentaries, and reviews) were excluded.

### Search outcome

The initial database search generated records from which articles were initially identified through screening of titles and abstracts as potentially relevant (LB and BR). Following removal of duplicates, papers of full text were read by two authors (LB and BR) to determine eligibility for inclusion. Discrepancies about whether a paper met the inclusion criteria were discussed with a third author (JSL) and a final decision was based on consensus. References of the full text articles assessed for eligibility were hand-checked to identify further papers that satisfied selection criteria.

### Data extraction and analysis

The following data from included papers were extracted: author, date and location of study, aim, sample, design and methods, data collection and analysis, and results.

Data from included studies were systematically extracted using a standardised tabulated form (Table 4) by LB and BR independently, and then discussed and combined. In order to address the aims of this review, data were extracted on the results from health-related quality of life measures and patient-reported outcome measures. For qualitative studies, extracted data were compared across studies and grouped into themes to describe the issues pertinent to PCD patients.

### Quality appraisal

Quality appraisals of data from both the qualitative and qualitative studies were independently assessed by LB and BR. The criteria for assessing the quality of quantitative studies as previously used by researchers [13–15] included study design, participants and recruitment, comparison group, number of participants, and quality of instrument used (Table 1). The total quality score ranged from 0 to 15 with each of the five criteria being score from 0 to 3. Quality assessment on the qualitative studies was performed using the Consolidated Criteria for Reporting Qualitative Health Research [16].

**Table 1:** Criteria for rating methodological quality of quantitative studies

Study parameter	Rating	Criteria
Study design	3	Longitudinal prospective design (explicitly stated)
	2	Retrospective or mixed design (explicitly stated)
	1	Cross-sectional (explicitly stated)
	0	Survey or did not report
Participants and recruitment	3	(1) Description of the population, (2) eligibility of participants, (3) precise details of the recruitment process, (4) accounted for the number recruited, (5) loss to follow up
	2	Minimal description of at least four criteria
	1	Two criteria missing
	0	More than two criteria missing
Comparison group	3	Healthy, age-appropriate comparison (i.e. adolescents/young people aged 13–25 years)
	2	Reference sample
	1	Other comparison group (i.e. adults)
	0	No comparison group
Number of participants	3	*n* > 100
	2	*n* = 50–100
	1	*n* < 50
	0	Did not report
Instruments used	3	Psychometrically sound report of instruments used
	2	Some weak psychometric properties reported
	1	Psychometric properties of instruments reported as inadequate for measuring HRQoL or IQ, physical functioning. etc
	0	No psychometric properties reported

Adapted from previously reported studies [13–15]

## Results

### Study selection

**Fig. 1:**
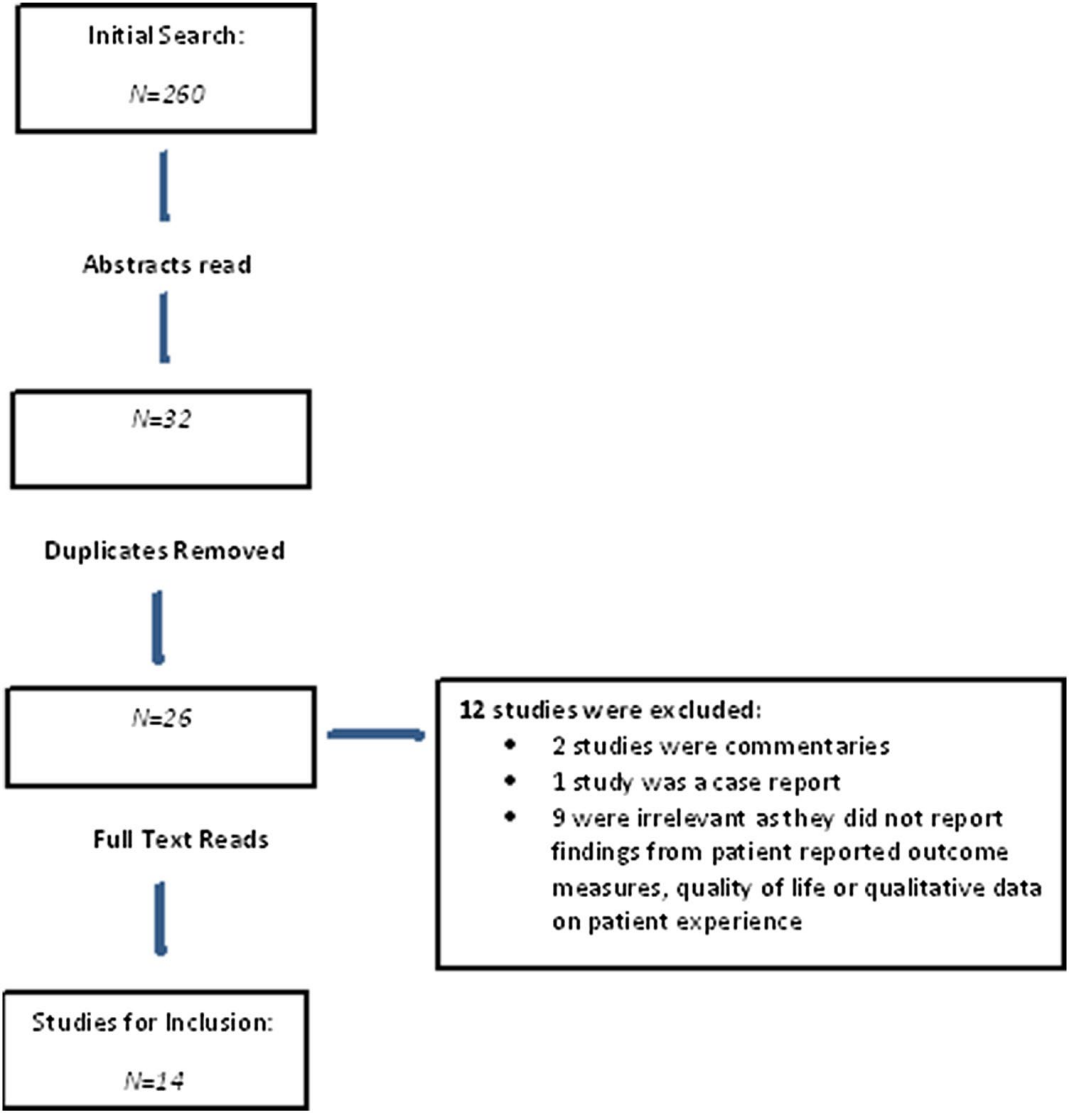
PRISMA Flow Diagram for search to investigate PCD from the patients’ perspective

The initial database search generated 260 records from which 32 articles were initially identified through screening of titles and abstracts as potentially relevant (Fig. 1). Removal of duplicates resulted in 26 papers of full text. Fourteen papers were identified for inclusion, two of which were conference abstracts where the full results were not available. For two additional abstracts (manuscripts now published) [10, 17], the authors had access to study results. No further papers were identified where references of the full text articles were hand-checked.

Studies included samples from the UK [11, 18–20], Italy [21–24], Denmark [25], and Belgium [26, 27] with collaborative works including participants from the UK and North America [17, 28] and an international study including participants from 25 countries [10]. Six studies consisted of cross-sectional surveys, two of which compared the PCD sample results with reference population norms [11, 20–22, 26, 27], one was a longitudinal survey given at two time points [23], three were case–control design including healthy samples for comparison [24, 25]. Four used a qualitative approach [17–19, 28] and one used a mixed method approach [10]. Three of the UK studies [11, 19, 20] were carried out using the same study population. The two Belgian studies were also conducted on a shared sample. Sample sizes ranged from 5 to 270. Apart from one study, gender was reported and all contained both male and female samples (Table 2).

**Table 2:** Summary of the 14 studies included in this systematic review, including the aims, study design, analysis, and findings

Study, Year, Country	Aims	Sample	Design	Data collection and analysis	Findings
Behan et al. [10] International	Investigate patient opinions about the PCD diagnostic process internationally	– Survey: 270 PCD patients from 25 countries. – Age: not specified – Gender: 114/271 males – Interviewed 20 parents/patients – Gender: 6/20 males – Age: not specified	Survey, cross sectional and semi-structured interviews	– A patient survey was developed by patient representatives and healthcare specialists to capture experience – Information collected included age, gender, age at diagnosis, time since diagnosis, diagnostic procedures, number of visits to GP before referral, questions relating to patient’s perception on diagnostic process – Semi-structured interviews were conducted and fully transcribed and thematically analysed	– 35% of respondents visited their doctor > 40 times with PCD related symptoms prior to referral for testing – Lack of PCD awareness among medical practitioners and failure to take past history into account leading to a delayed diagnosis – In the diagnostic process, improved reporting of results and a solution the ‘inconclusive’ diagnostic status were considered as needs – A significant difference was found between those who were diagnosed in childhood (0–12 years) and those who were diagnosed in adolescences/adulthood (>13 years) on the level of agreement that health has improved since diagnosis (*p* = 0.041) – Difficulty getting antibiotics, and isolation due to poor communication between GPs and specialists was reported after diagnosis
Carotenuto et al. [24] Italy	To perform behaviour and psychological evaluation of children with PCD compared to controls of healthy children. To assess if PCD effects and impacts the quality of family functioning and the psychological equilibrium of children	– 10 PCD and 34 healthy school-aged children – PCD: 7/10 males – PCD Age range: 8–16 years – Healthy: 24/34 males – Healthy Age range: 6–16 years	Survey; case-control, cross-sectional	– Standardized questionnaires – Children completed Wechsler Intelligence Scale – Parents completed Child Behaviour Checklist questionnaire and Parenting Stress Index-Short Form	– No significant differences between age, gender or BMI, or mother’s age and educational level between the 2 groups. – No difference in IQ (WISC-III) between the two groups. – For CBCL no clinical relevant scores were found for both group – Higher scores were found in the PCD group compared to the healthy group for withdrawn, somatic complaints, anxious/depressed items, attention and internalizing problems items (*p* < 0.05). – Parent Stress Index Short-Form mean scores relating to parental distress, child parent interaction and total stress in mothers was significantly higher in the PCD group – All PCD mothers had high levels of stress.
Dell et al. [17]^b^	To develop harmonized (North America, Europe) paediatric HRQoL questionnaires for children (6–12 years), adolescents (13–18 years), and parent respondents	Age range: 6–17 years Gender: 20/40 males	Focus groups and open-ended interviews	– Literature review, focus groups (clinician and patient) and semi-structured interviews with children, adolescents and their parents – Transcripts were content-analysed – Item relevance survey – Questionnaires refined following cognitive interviews	– This led to the development of four age-specific preliminary instruments measuring HRQoL in PCD patients – These consist of a: (1) Child version (6–12 years) (37 items); (2) Adolescent version (13–17 years) (43 items); and (3) Parent version (children aged 6–12 years) (41 items) – Measures consisted of 8–10 scales including: Impact to Physical, Emotional and Social functioning, Vitality, School Functioning, Lower and Upper Respiratory Symptoms, Impact of ear symptoms/hearing loss, Impact of treatment burden, Impact to Eating and Weight
Lucas et al. [28] UK and North America^c^	To develop a PCD-specific HRQoL instrument for adults with PCD	– 21 PCD adults – 3/21 males – Age range: ≥18 years	Focus groups and open-ended interviews	– Open-ended interviews – Content analysis yielded the most important items for each of the 10 domains based on the frequency with which they were mentioned. Saturation confirmed when no new themes emerged – Item relevance survey – Questionnaires were refined following cognitive interviews	– 10 domains based on frequency with which they were mentioned across adult group – Cognitive interviews provided 6 additional items. – The final prototype instrument contained 49 items across ten domains and included impact of respiratory symptoms, impact of sinus symptoms, impact of ear symptoms/hearing loss, impact to physical functioning, impact to emotional functioning, impact to social functioning, vitality, health perception, role functioning, impact of treatment burden
Madsen et al. [25] Denmark	To assess peak oxygen uptake (VO_2_peak) to compare these values with those of healthy subjects; and assess if VO_2_peak is associated with parameters of pulmonary function, self-reported physical limitations, and weekly physical activity	– 44 PCD adults and children matched with 33 healthy controls. – PCD: 17/44 males – PCD age range: 6.0–29.7 years. – Healthy: 17/33 males – Healthy age range: 6.2–28.8 years	Survey, cross-sectional, case control study	– 3 questions about physical activity and limitations were extracted from standardized questionnaires; St George’s Respiratory Questionnaire, Cystic Fibrosis Questionnaire, Sino-Nasal Outcome Test-22 and the Medical Outcomes Study Short Form-36	– In response to questions relating to physical activity, 34% of patients reported being moderate to highly limited, 44% were slightly limited while 21% were not limited at all by sino-pulmonary symptoms in activities of everyday life. – 39% reported moderate to severe limitation in performing vigorous activities, while 30% only reported only slight difficulties and 30% denied having any difficulties. – VO_2_peak was significantly lower in patients reporting severe limitations in performing vigorous activities compared to patients without limitations. – VO_2_peak was significantly lower in patients who reported being highly limited by sino-pulmonary symptoms in everyday life compared to patients who were not limited at all.
Maglione et al. [23] Italy	To verify HRQoL in respiratory disorders correlate with spirometry or a 6-min walk test	– 20 PCD patients – Gender not specified. – Age range: 12.0–33.4 years	Survey, longitudinal	– Standardized questionnaires were completed by patient: St George’s Respiratory Questionnaire, Leicester Cough Questionnaire, Medical Outcomes Study Short Form 36 at two time points – Data on spirometry and the 6-min walk test at 2 time points also collected	– Spirometry and 6 min walk test were not significantly related to any of the HRQoL assessment tools at baseline or 12 months later. – Over the 12 month period, no significant changes were found in any of the HRQoL outcomes or in spirometry of 6 min walk test. – HRQoL tools used reported as suboptimal to longitudinally track HRQoL in PCD
McManus et al. [11] UK^a^	To examine the effect of PCD on the overall health status of patients	– 93 members of UK PCD Family Support Group. – 34/93 males. – Mean age = 22.7 (SD 16.8) and median 16.5 (IQR 10.8–31.3)	Survey, cross-sectional	– Standardized questionnaires were completed by patient; St George’s Respiratory Questionnaire and Medical Outcomes Study Short Form 36 – Separate versions of the questionnaire were provided for adults and children (< 16 years)	– SGRQ domains; Symptoms, Activity and Impact scores correlated significantly with age and declined more rapidly after 25 years and more rapidly than population norms. – SGRQ domain: Impact and Activity show effect of time since diagnosis – Almost all patients reported ‘a runny nose and nasal congestion’, ‘pain over my sinuses’ and ‘a headache’ affected patients a few days a month.
McManus et al. [20] UK^a^	To describe the influence of demographic factors, respiratory symptoms, physical and mental health status and stress upon stigma experienced by patients and their relationship with the Big Five measures of personality	– 71 members of UK PCD Family Support Group – 23/71 males – Mean age = 27.7 (SD 16.2) and median 20.1 (IQR 15.6–38.7) (Only respondents’ ≥10 years were included.)	Survey, cross sectional	Standardized questionnaires were completed: St George Respiratory Questionnaire, Medical Outcomes Study Short Form 36 General Health Questionnaire, personality (‘Big Five’). Stigma using the authors own questionnaire was also. Separate versions were provided for adults and children (under 16 years)	– Stigma had no association with age or age of diagnosis – It correlated significantly with the SGRQ Symptom and Impact of Illness Score but not with Activity Score. – Stigma correlated with the GHQ stress score and with the Mental Summary of the SF-36 although not the Physical Summary scores. – The stigma score correlated with neuroticism measure – Stigma did not differ between males and females or between those with situs inversus and situs solitus. – High Impact measure on the SGRQ and good mental health (SF-36) and Low Activity (SGRQ) are predictors of Stigma
Mirra et al. [22]	To investigate if levels of Vitamin D are associated with quality of life and self-reported activity level, among other outcome parameters	– 22 PCD patients, – 15/22 males. – Age range: 2–34 years	Survey, cross-sectional	– Standardized questionnaires were completed: St George’s Respiratory Questionnaire and Self-reported Physical Activity levels. – Patients underwent serum vitamin D levels measurement, pulmonary function tests, deep throat and sputum culture	– SGRQ score was 19 (9–65). – For physical activity, 10% of patients reported moderately-to-highly limited, 26% were slightly limited and 63% were not limited at all by respiratory symptoms in everyday activities. – 52% of cases reported moderate-to-severe limitations in performing vigorous activities, while 26% had only slight difficulties, 21% had no difficulties at all.
Pifferi et al. [21] Italy	To assess the impact of PCD on HRQoL in Italian patients. To identify the unmet needs of the patients and the potential diagnostic and therapeutic pitfalls	– 78 PCD patients – 34/78 males – Age range: 1.7–48.5 years	Survey, cross-sectional	– Standardized questionnaires were completed: St George Respiratory Questionnaire, Medical Outcomes Study Short Form-36. – Information on age, gender, age at diagnosis, time since diagnosis, clinical features, compliance with treatment, diagnostic procedures, incidences of surgery in patients, PCD in family members also collected. – A questionnaire on clinical course of the disease management, including a question on the patient’s perception on quality of life after diagnosis was completed. – Separate age specific versions used.	– All 3 subscales of SGRQ correlated with age – Cough on almost all days a week was the most frequent reported symptoms (48.7% of patients). – Significant correlation between time since diagnosis and impacts subscale but not for symptoms subscale (SGRQ). – Breathlessness increased with age. – There was a decline in physical and mental component scores (SF-36) in relation to age in PCD patients, significant only for mental component scores. – Age at diagnosis influence on symptoms, activity and impacts (SGRQ) and mental health (SF-36) – Reduced compliance with treatment is associated with mental component scores (SG-36) and age at diagnosis and time since diagnosis – The majority (71.8%) considered their quality of life to have significantly or slightly improved after diagnosis
Schofield et al. [18] UK	To explore the physiotherapy experiences of patients and their parents within the paediatric PCD population in the UK. To identify patients’ needs and to make recommendations for future service developments	– 3/5 males – Age range: 8–15 years (all Asian ethnicity)	Semi-structured interviews	– Interpretative phenomenological analysis. – Pilot interview conducted: themes based on concepts from existing literature – Subsequent interviews involved the participant recounting their daily routine – A second validation interview discussed key points of the first interview	– Experience of day to day life with symptoms and treatment burden – Diagnosis led to symptoms perceived as abnormal – Symptoms reduced since treatment began. Coughing was variable in its acceptance and depended on severity – Embarrassment from coughing. Revulsion from coughing up sputum. Anxiety looking towards the future and how long-term improvements could be sustained fuelled anxiety. Freedom emerged from being able to engage in activities without limitation – Participant’s self-awareness and self-assessment of symptoms. Knowledge of condition and the preventative nature of physiotherapy varied. Limited sharing of PCD with teachers and peers and even at home – The role of the family, carers and health specialists in nurturing personal mastery skills. Clinics provide knowledge and treatment skills which were then refined into practices that were personally enjoyable and effective.
Taelman et al. [26] Belgium^b^	This study aims to investigate and identify attitudes and barriers related to treatment adherence in children with PCD and their parents	– 25 parents of PCD children (<18 years) – 7 PCD adolescents – Age range: 14–18 years	Survey, cross sectional	– A questionnaire consisting of demographic information and treatment related questions – A list of 18 barriers and 10 statements of attitudinal patterns – Adolescents completed questionnaire independently	–The most commonly reported barriers to treatment were too busy, forgetting, family issues, wanting to be normal, takes too much time – For adolescents, attitudes influencing non-adherence include PCD team does not understand how tough it is to follow treatments (57.1%), wanting to follow my treatments but sometimes just forget (71.4%), trouble sticking to treatments because they make teenager feel worse (85.7%), having to follow treatments means less freedom in life (42.9%).
Taelman et al. [27] Belgium^b^	To examined the impact of PCD on daily life by comparing self-reported and prescribed treatment; investigating barriers and attitudes to treatment and exploring coping styles	– 39 PCD patients (25 parents) – 13/39 males – Mean age = 33 years	Survey, cross sectional	– A questionnaire consisting of treatment related questions – Age, gender, FEV1, types of treatment completed also completed	– Frequency of treatments varied with 82% parents reported daily use of nebulizer; 64% patients reported daily use of nose spray and 46% reported physiotherapy – Agreement between self-reported and prescribed treatment ranged from 39% for eardrops to 71% for antibiotics and 89% for physio – Most patients (96%) did not agree that their health will be OK, even if treatments are not done and parents (76%) agreed that having to follow treatments means less freedom in life – Burden of treatment is related to time and wanting a normal life
Whalley et al. [19] UK^a^	Depth-qualitative interviews aimed to explore themes surrounding the psycho-social impact of PCD. A quasi -experimental design was used for directly validating the stigma questionnaire	– 6 pairs (*n* = 12) of PCD patients. – 2/12 males. – Aged range: 27–65 years	Depth qualitative interviews followed by stigma rating	– Grounded theory analytical approach – Interviews conducted and fully transcribed. – Initial themes under investigation included diagnosis, symptoms and social perspectives surrounding PCD, including the possibility of stigma – Before each interview, the previous was transcribed and loosely open-coded, with emerging themes compared with the previous interview data – Comprehensive open-coded once data collection was complete	– Other people’s lack of knowledge of PCD led to frustration in some but other understood this was due it being a rare disease. Some educate others and are open. Others were more censored avoiding describing the stigmatized symptoms such as productive cough. Some were under pressure to disclose while others at some point avoided disclosure particularly when at school. – Most had at some stage tried to conceal symptoms – Embarrassment from symptoms led to behavioural change – Failure to diagnosis PCD until later in life left some feeling mistrust of medical care. Mistrust in GPs; difficulty getting antibiotics and isolation due to poor communication between GP and specialist. However praise of tertiary specialist centre – Ratings of stigma scales were in complete concordance

^a^Same UK study population

^b^Same Belgian study population

^c^Both publication are part of the same study with UK and US participants; however, different age groups, therefore separate study populations

### Methodological quality

Quality appraisal of the quantitative studies points to deficits, in particular to study design, and recruitment and inclusion of a comparison group (Table 3). Psychometric properties of the measures were cited in five of the quantitative studies; however, the internal reliability of the measures, i.e. Cronbach’s alpha within the sample population, was not reported in any of the studies. The application of the measures was not clear for all studies. For some studies, measures used were not developed/adapted and validated specifically for younger children. Most studies were surveys (with one being longitudinal) and although it was apparent that these studies were cross-sectional, this was not stated explicitly in all. The study which ranked the highest had a score of 8 out of 15 points; this was a cross-sectional survey study where 78 patients completed a questionnaire which collected information on age of diagnosis, symptoms and likely PCD-specific problems in addition to disease-specific and general HRQoL (using the St. George’s Respiratory Questionnaire and the Medical Outcomes Study Short Form-36 (SF-36)). Use of the SF-36 allowed for scores to be compared with the healthy Italian population.

**Table 3:** Summary of the quality of the data and studies contributing to the quantitative studies included in this systematic review

	Study design	Participants and recruitment	Comparison group	Number of participants	Instruments	Total
Behan et al. [10], International	0	2	0	3	0	5
Carotenuto et al. [24], Italy	0	0	3	1	2	6
Madsen et al. [25], Denmark	1	2	3	1	0	7
Maglione et al. [23], Italy	3	0	0	1	0	4
McManus et al. [20], UK^a^	0	1	0	2	2	5
McManus et al. [11], UK^a^	0	1	2	2	2	7
Mirra et al. [22]	1	0	0	1	2	4
Pifferi et al. [21], Italy	1	1	2	2	2	8
Taelman et al. [27], Belgium^b^	0	0	0	1	0	1
Taelman et al. [26], Belgium^b^	0	1	0	1	0	2

Scoring was according to Criteria for Rating Methodological Quality of Quantitative Studies adapted from previous studies [13–15]

^a^Same UK sample n

^b^Same Belgian sample

For the four qualitative studies, criteria of the COREQ-32 item checklist are generally adhered to Table 4. The main deficits in reporting were the characteristics of the research team and the relationship between interviewee and interviewer, description of the coding tree, and the provision of feedback to the interviewee.

**Table 4:** Summary of completeness of reporting for the qualitative studies included in this systematic review using the Consolidated Criteria for Reporting Qualitative Health Research [16]

Reporting criteria	No (%) *n* = 5	References of studies reporting each criterion
Characteristic of research team		
Interviewer/facilitator identified	4 (80%)	[23, 24, 35, 36]
Credentials	2 (40%)	[23, 35]
Occupation	2 (40%)	[23, 35]
Gender	0 (0%)	–
Experience and training	2 (40%)	[23, 35]
Relationship with participants		
Participation knowledge of the interviewer	2 (40%)	[23, 35]
Interviewer characteristics	3 (60%)	[23, 24, 35]
Methodological orientation and theory	5 (100%)	[23, 24, 34–36]
Participant selection		
Sampling method (for example, snowball or purposive)	5 (100%)	[23, 24, 34–36]
Method of approach	5 (100%)	[23, 24, 34–36]
Sample size	5 (100%)	[23, 24, 34–36]
Non-participation	2 (40%)	[23, 24]
Setting		
Setting of data collection	4 (80%)	[23, 34–36]
Presence of non-participants	1 (20%)	[23]
Description of sample	5 (100%)	[23, 24, 34–36]
Data collection		
Interview guide	5 (100%)	[23, 24, 34–36]
Repeat interviews	1 (20%)	[23]
Audio/visual recording	5 (100%)	[23, 24, 34–36]
Field notes	3 (60%)	[23, 24, 36]
Duration	2 (40%)	[23, 24]
Data saturation	4 (80%)	[23, 34–36]
Transcripts returned to participant	0 (0%)	–
Data analysis		
Number of data coders	5 (100%)	[23, 24, 34–36]
Description of the coding tree	0 (0%)	–
Derivation of themes	5 (100%)	[23, 24, 34–36]
Protocol for data preparation and transcription	5 (100%)	[23, 24, 34–36]
Software	4 (80%)	[23, 34–36]
Participants’ feedback or member checking	3 (60%)	[23, 34, 35]
Reporting		
Participant quotations presented	5 (100%)	[23, 24, 34–36]
Data and findings consistent	5 (100%)	[23, 24, 34–36]
Clarity of major themes	5 (100%)	[23, 24, 34–36]
Clarity of minor themes	5 (100%)	[23, 24, 34–36]

### Methodologies of quantitative and qualitative studies

Six studies assessed health-related quality of life (HRQoL) in PCD patients. HRQoL measures are generic or disease-specific. Disease-specific measures assess special states and concerns of different diagnostic groups and are important for the detection of small clinically important changes. The most commonly used disease-specific HRQoL measure in this review was the St. George’s Respiratory Questionnaire (SGRQ) for chronic obstructive pulmonary disease (*n* = 6). Other disease-specific outcome measures used included the HRQoL measure for cystic fibrosis (CFQ-R) (*n* = 1), a HRQoL measure for sinonasal conditions: The Sino-Nasal Outcome Test (SNOT-22) (*n* = 1), and the Leicester Cough Questionnaire (LCQ) (*n* = 1). To assess the impact of PCD on HRQoL, related to mental health and well-being, the Medical Outcomes Study Short Form 36 (SF-36) was used in five of the studies. Other patient-reported measures that measured outcomes other than HRQoL included the Wechsler Intelligence Scale for Children, the Child Behaviour Checklist questionnaire, the Parenting Stress Index–Short Form and the Self-reported Physical Activity Measure. One study included a questionnaire measuring Stigma [20]. This was developed by the author and stigma was assessed by the patient’s response to 11 items on embarrassment about symptoms, feeling a nuisance to friends or family, concealment of condition, etc. Four of the studies were cross-sectional, single-occasion, single-centred studies. One study was a cross-sectional, single-occasion, single-centred case–control study, and one was a cross-sectional, longitudinal, single-centred study with measures repeated after 1 year.

### Main themes

#### Factors influencing health-related quality of life

In a cross-sectional UK survey [11], a slight decline in HRQoL was found for all three domains of the SGRQ (Activity, Impacts and Symptoms) until the age of 25 years after which a more rapid decline occurred. The physical component score of the SF-36 also showed a continual decline with age so that by the age of 40 onwards, the health status of PCD patients was one and a half standard deviations below the population mean. In contrast, the mental component score also declined with age however the declining health status broadly parallels that found in the general population as a whole, and was, at the most, one-third to one-half a standard deviation below the population norms. Age was also an important factor in an Italian cross-sectional survey study [21], where all three subscales of the SGRQ and the physical and mental component scores of the SF-36 declined significantly greater than norms for the corresponding Italian population. These declines, however, were found to be earlier in age than those reported in the UK study [11], where deterioration mainly occurred prior to and during adolescence. In the UK study, little abnormality was found for the childhood and adolescence study population when compared to standard measures of the SF-36.

Both studies found that patients with an earlier diagnosis had better scores for the SGRQ Impact and Activity subscales, suggesting the importance of early medical intervention for HRQoL. The Italian group found a clear majority of patients (71.8%) considering their quality of life significantly or slightly improved after diagnosis; however, there remained a progressive worsening of the disease over time. This was in contrast to the UK group who reported stable scores for patients after diagnosis.

#### Physical impact

Ten studies addressed the impact of PCD on physical functioning [10, 11, 17, 18, 20–23, 25, 28]. The physical impact of PCD was reported by children, teenagers, and their parents in a qualitative study using phenomenological analysis methods [18]. Coughing was regularly mentioned by all participants in their accounts of daily activities, as was the impact of their cough on activities when both well and unwell. Symptomatic relief of chest symptoms was reported as leading to a sense of freedom at being able to undertake activities without restrictions. Patients reported feeling limited in their ability to keep up with peers because of coughing, breathlessness, fatigue and low energy levels. Similar themes arose in two collaborative qualitative studies, which included interviews with patients from the UK and North America [17, 28]. Children and teenagers reported they became tired quickly when engaging in physical exercise and needed to request more breaks than their peers. This theme relating to the physical impact of PCD was also found in adult interviewees, where patients reported not being able to keep up with others when walking or exercising (Box 1).

**Box 1:** Patient experiences of the physical impact of PCD

A: “I go running again and then cough a bit and then I’ll stop” Child [18]
B: “I had to tell the group not to worry because I start huffing and spluttering as I’m walking.” Adult [28] “My air goes out because I’m running and I can’t speak and then I’m not speaking and sometimes my air goes down a bit and then I can’t, and then I just can’t, I can’t, I can’t take it.” Child [18] “…if he’s playing in school and …he needs to run around, then he gets more tired than other kids and they’re still running around and he’s stopping.” Parent [17]

In a quantitative study, 10% of patients were found to be moderately-to-highly limited by respiratory symptoms in everyday activities, and 52% of cases had moderate-to-severe limitations in performing vigorous activities [22]. This was in contrast to a Dutch study where 34% of patients reported being moderately-to-highly limited by sinopulmonary symptoms in activities of everyday life, and 39% reported moderate-to-severe limitations in performing vigorous activities. None of the healthy controls reported any limitations in physical abilities.

As reported previously, a continual decline according to age in scores on the physical domain of the SF-36 reflected a moderate degree of morbidity on normal physical functioning which is progressive across the lifespan [11]. Cough, on almost all days of the week in the last 12 months, was the most frequently reported symptom (48.7% of patients) regardless of age, together with excessive sputum (57.7% of patients) [21].

#### Emotional impact: frustration, anxiety and stress

The emotional impact was explored in depth in three of the five the qualitative studies [17, 18, 28]. Interviews in the UK and North America explored the emotional impact of PCD in all age groups [17, 28]. In the paediatric group, frustration relating to treatment burden was a prominent theme. Children and adolescents reported feeling frustrated about getting sick regularly and about the chronic nature of their symptoms. In addition, a sense of unfairness and sadness about having this condition was reported. A UK qualitative study [18] found that children and teenagers became anxious when thinking about their health in the future. The positive changes which had arisen from their diagnosis and effective health care, while appreciated, induced a level of doubt and anxiety as to how these improvements could be sustained. Such feelings of anxiety were also found in a series of interviews with adult patients [28]. This was especially the case when thinking about their future and future health. They reported feeling anxious about being able to conceive children as well as being well enough to care for their family (Box 2).

**Box 2:** Patient experiences of the emotional impact of PCD

A: “I was sick on and off…it’s just frustration. Because there’s no cure.” Adolescent [17]
B: “Sometimes, when he sees his friends running around and he can’t tag them, then he feels like *why do I have PCD?*” Parent [17]
C: “It…just wastes all of my energy, it makes me feel like I don’t want to wake up in the mornings” Child [17]
D: “I’m so frustrated with this illness, I just want it to go away, but, unfortunately, that’s how I have to live.” Adult [28]
E: “…if you go to the doctor [and] you’re feeling pretty good and you know your numbers are not good; that can be a big cause of anxiety.” Adult [28]
F: “Finding out that I possibly can’t have kids; that are when it started to panic me a little bit.” Adult [28]
G: “I’m still very uncertain if I ever wanna have children because I don’t know how me having this illness will affect them.” Adult [28]

Carotenuto et al. conducted a behavioural and psychological evaluation of children with PCD and compared the results to healthy children [24]. The findings showed no clinically relevant scores for both healthy and PCD groups. However, higher scores were found in the PCD group for factors such as withdrawnness, somatic complaints, anxious/depressed items, attention span, and internalising problems items (*p* < 0.05). This study also found that total stress levels [assessed through the parenting stress index–short form (PSI/SF)] in mothers were significantly higher in the PCD group than in mothers of healthy controls (*p* < 0.01), and that all PCD mothers had high levels of stress.

#### Social impact: Stigma, embarrassment and concealment

In the qualitative studies, symptoms such as coughing, sputum production, and ear drainage were reported as causing embarrassment among paediatric patients [17, 18, 28]. Acceptance of coughing was found to be variable among participants and depended on severity. There was also a sense of revulsion from coughing up sputum. Symptom relief led to patients feeling ‘normal’ [18] (Box 3), paradoxically a reluctance to adhere to treatments was also attributed to wanting to feel normal [26, 27]. Adult patients also reported embarrassment [19], with patients concealing symptoms such as coughing and blowing their nose. In a study assessing stigma (measured using a questionnaire developed for this study) [20], 75% of the sample agreed that their coughing or breathing was embarrassing in public. It also found that stigma correlated with symptoms and impact of illness from the SGRQ but not with activities. It also correlated with the mental health component scores of the SF-36 but not for the physical component scores.

**Box 3:** Patient experiences of the emotional impact of PCD

A: “actually coughing up mucus isn’t a very nice thing. It’s not, it’s quite a sort of...frowned on in society kind of thing isn’t it so I kind of, yeah, I don’t think it’s very nice, sort of, to do it in front of people” Adult [19]
B: ‘Sometimes I raise my hand and then say, ‘I have to blow my nose.’ And then I go in the bathroom…and shut the door because I don’t want anyone to hear me [because] it’s embarrassing.” Child [17]
C: “I feel like I’m being judged by other people because I constantly sniff and…cough.” Teenager (Dell)
D: “If she has a speech problem or…coughing constantly…when they’re in school, it might become embarrassing.” Parent [17]
E: When I cough. .. it feel a bit more, erm.. . like I’ve got PCD, but when I don’t cough I just feel normal. Child [18]

Paediatric patients were found to be reluctant to share their PCD diagnosis with teachers and peers or even to talk about their condition at home [18]. In a separate UK study of patient ≥10 years, 45% of patients agreed in a study-specific questionnaire that they have sometimes felt they had to hide their condition from other people [20]. Following on from this, a qualitative study [19] found that some patients felt frustrated by lack of knowledge of PCD in the general public. While some interviewees were keen to educate others and were open to discuss their illness, others were more censored, and avoided describing their symptoms. The likelihood of disclosure may be dependent on context, since some patients felt under pressure to disclose their diagnosis, for example, to teachers or work managers on an account of needing time off when ill. There were other patients who reported avoiding open disclosure, particularly when at school (Box 3).

#### Lack of PCD awareness among medical practitioners

A mixed method study [10] reported the accounts of 20 adult patients and parents of children and teenagers from nine different countries on their experience of being diagnosed with PCD or going through the diagnostic process. The most prominent theme reported among interviewees was a frustration with the lack of PCD awareness among medical practitioners, manifesting initially in the failure of general practitioners (GPs) to refer them for PCD diagnostic testing. This was also found in a UK-based qualitative study, using grounded theory analytical methods [19] where failure to diagnosis PCD until later in life left some patients feeling distrustful of medical care. Themes such as distrust in GPs, difficulty getting antibiotics, and isolation due to poor communication between GPs and specialists were reported by both studies [10, 19].

#### Treatment adherence and treatment burden

Two abstracts [26, 27] from a survey using the same sample but presenting separate results examined treatment adherence. A range in the levels of agreement was found between self-reported and prescribed treatment, ranging from 39% for eardrops, to 71% for antibiotics, and 89% for physiotherapy. Barriers to completing treatments included being too busy, forgetting about treatments, family issues, and treatments taking too much time. For adolescents, 57% agreed that their PCD team do not understand how difficult it is to follow treatments, and 43% felt that having to follow the PCD treatments meant less freedom in life. The difficulty of fitting treatments in on a daily basis was reported by 12/20 adolescents interviewed across the UK and North America [17]. Interviews with adult PCD patients also reported the challenges of completing their treatments [10, 28] (Box 4).

**Box 4:** Patient’s experiences of treatment burden

A: “I think it just requires more planning. I need to wake up earlier or start getting ready for bed earlier, I need to come home from work and do this; it’s just more planning.” Adult [28]
B: “It was a bit of a shock…. I was probably in my mid-thirties then, to suddenly be told, right, you’ve got to do 20 minutes of physio twice a day, you’ve got to take this blue puffer, and the brown puffer… as soon as you get a chest infection you’ve got to take really strong antibiotics, I rebelled against that” Adult [10]
C: “She was sick every month. Once we had a diagnosis… she gets sick, but not as severe as… before.” Parent [10]
D: Definitely milder…you know we have a treatment plan and even when she starts to get sick; those medications are changed so we tend to catch that right away rather than after that.” Adult [10]

There was agreement among parents of children with PCD (76%) that barriers to completing treatments meant less freedom in life [26]. Parents expressed how other commitments, such as siblings and employment, could limit their ability to complete daily treatments [17, 18] (Box 4).

Patients did report that following a PCD diagnosis, treatments could reduce symptoms providing sensations of relief. There was a subjective perception of physiotherapy treatments, corresponding to fluctuating levels of motivation. There was also a variance different levels of PCD health literacy knowledge in the preventative nature of physiotherapy among children and teenagers [18]. In a cross-sectional survey study [26], 86% agreed that they had difficulty complying with treatments because they made them feel physically worse; however, 96% of patients acknowledged their health would decline without treatments. In the mixed methods study by Behan et al., a study-specific survey [10] found a significantly higher level of agreement that health had improved since diagnosis (*p* = 0.041) in those diagnosed in childhood (0–12 years) compared to those who were diagnosed in adolescences/adulthood (>13 years).

## Discussion

This systematic review identified 14 studies focussing on the perspectives, opinions, and attitudes of patient with PCD. Most of the quantitative studies consisted of small cross-section surveys and the methodological quality of these studies was generally low (Table 3). While the qualitative studies provided a deeper insight into the patient experience, only a small number of these studies exist, and mostly include patients from the UK and North America. Notwithstanding these weaknesses, the evidence assembled from the studies makes an important contribution to understanding the PCD patient experience and associated influences relating to quality of life.

Two cross-sectional studies suggested a correlation between age and worsening of respiratory symptoms, general physical and mental quality of life. Within these two studies, variances exist with Pifferi reporting an early decline in HRQoL and McManus reporting little abnormality in standard measures of SF-36 during childhood and adolescence. Also the variances between the two studies could be due to differences in the age of participants involved or a result of their limited sample size. It could also be due to cultural differences between the countries (UK and Italy), access to specialist diagnostic, and management services or treatment adherence may also account for differences. Caution must be exercised in the interpretation of these findings. Cross-sectional studies do not take into account confounding factors such as differences between adult and child participants and experience which may affect changes over time, i.e. diet, tobacco smoke exposure, etc. The progressive nature of PCD and the deterioration of health have been described in other studies through physiological methods such as spirometry [5]. Werner et al. [29] have shown the percentage-predicted forced expiratory volume in 1 s (FEV1% pred) values versus age exhibited a mean annual decline of 0.59%. The results show interesting trends and however highlight the need for large longitudinal international studies before more reliable conclusions can be made. The Genetic Disorders of Mucociliary Clearance Consortium (GDMCC), the iPCD cohort and the BESTCILIA registry are examples of ongoing large-scale studies that will contribute to this aim.

The physical impact of PCD was a prominent theme in both the qualitative and quantitative studies. This was defined by the most prominent feature of this illness: coughing. Coughing was regularly mentioned by interviewees of all age groups [17, 18, 28]. It was the most frequently reported symptom in a survey of 78 participants, where 48.7% reported having to cough nearly all days of the week for the past 12 months [21]. Persistent presence of cough was found to be far less prevalent than that in other studies [10, 30] where it was found to be as high as 93–100%. This could be as a result of the way in which the question was phrased or the method of data collection used, i.e. patient reporting at home or reporting to a clinician in a hospital setting. Severity of symptoms might also reflect different data collection points, with patients on their first referral appointment prior to diagnosis and commencement of treatments exhibiting more severe symptoms. The physical impact of PCD was expressed by patients in the qualitative studies, as not being able to keep up with other family members and peers due to fatigue [17, 18, 28].

Questionnaire findings [24] showed that PCD children were more likely to be withdrawn, experience anxiety or depression, and internalise more problems than the healthy population. PCD was found to affect the parent also with significantly higher stress being reported in mothers of children with PCD. No other PCD study reports on these factors; however, studies in children and parents with cystic fibrosis have also reported elevated levels of depression, stress and anxiety compared to healthy populations [31, 32]. The synthesis of the qualitative studies allows the researcher to conclude possible reasons for this. PCD impacts greatly on the emotional functioning of patients in all age groups. Children described the frustration of having constant symptoms and recurrently getting sick. Patient anxiety was expressed, especially when thinking about the near and distant future. Children reported feeling worried about their health and of getting sick. A sense of sadness was reported because of their awareness of being different from other children. There is a need for further exploration on how PCD causes stress in developmental ages and the psychological effects of PCD on intra-familiar relationships.

Concealing PCD symptoms such as cough and blowing nose in public were reported across the qualitative studies [17–19, 28]. Embarrassment was mostly from coughing and producing sputum in public; however, ear drainage was also reported as an embarrassing symptom in one of the paediatric studies [17]. The stigma questionnaire (which included items on embarrassment from symptoms and concealment) correlated with mental health and the social impact of symptoms. Although the impact of PCD on school functioning was expressed by patients [17, 28], no differences in educational level or IQ were found between PCD children and healthy children. School functioning instead could be related to patients’ reluctance to disclose their PCD diagnosis with teachers and peers. Such concealment of symptoms and illness disclosure has been reported across chronic illness [33–36]. Results from a cystic fibrosis study [36] found that patients were more likely to disclose to romantic partners and close friends than to casual friends, bosses, or co-workers, and disclosure was associated with higher social support, social functioning, and medication adherence self-efficacy.

Poor adherence to treatments can often be a conscious decision in PCD; however, it can be the result of not making any decisions at all, e.g. worry about having PCD could lead to attempts to avoid thinking about it. Poor adherence however is likely to lead to raised anxiety about the consequences, which often leads to attempts by the individual to minimise the risks [37]. This process is known as cognitive dissonance which refers to the widespread observation that in any situation where people who feel uncomfortable about a choice they have made, also hold a strong desire to resolve this discomfort. Its resolution is central to motivating patients to change [38]. Cognitive dissonance has been reported in cystic fibrosis; however, further investigation is needed in PCD. This literature synthesis did find that symptom relief led to patients feeling ‘normal’ [18] but paradoxically, there was a reluctance to adhere to treatments which was also attributed to wanting to feel normal [26, 27]. Furthermore, in a cross-sectional survey study [26], 85.7% agreed that they had difficulty complying with treatments because they made them feel physically worse; however, 96% of patients acknowledged their health would decline without treatments. There was also a variance in the levels of PCD health literacy and in the knowledge of the preventative nature of physiotherapy among children and teenagers [18]. The perception of physiotherapy treatments, which corresponding to fluctuating levels of motivation, highlighted the need for patient centeredness and personalised medicine.

### Limitations

The review has limitations. Papers included were limited to those published in the English language. It is possible that there are relevant studies published in other languages. Overall the evidence of this review is based on a small number of heterogeneous studies (*n* = 14) that are limited in size. The quality assessment of the quantitative studies revealed them to be of low quality with scores no greater than 8 points. Until recently, no disease-specific age-appropriate HRQoL measures were available for PCD patients [17, 28] and to date, studies have used general HRQoL tools such as the SF-36 and disease-specific tools for cystic fibrosis and COPD. These studies have also included child participants to complete measures that are not age appropriate without psychometric validation. Studies have included results where young children had help from a parent to complete these measures which may lead to bias [34]. Only one of the studies performed analyses with and without the children who needed help completing the questionnaire. In addition, limited psychometric data were presented on the validity of the HRQoL used, with some studies reporting validity but never for all of the scales. As with any review, the quality of studies included can only be assessed by what was reported in the final manuscript, e.g. missing information on any of the adopted criterion might reflect unclear reporting as opposed to a limitation in study design.

### Recommendations

To date, no medications to treat PCD have been approved by regulatory bodies [30] and current physiological outcome measures such as spirometry, chest computed tomography, and lung clearance index have been reported to have limitations in terms of their sensitivity and feasibility for evaluating new therapies or disease progression [4, 39–41]. These physiological measures also do not reflect the impact of the disease on patients’ daily symptoms or functioning (e.g. physical, respiratory, social) as required by the Food and Drug Administration [42] and the European Medicines Agency [43, 44]. This study has highlighted the need for large multi-national and longitudinal studies to be conducted using PCD-specific health-related quality of life measures (QOL-PCD) [28]. Studies are underway and QOL-PCD has been developed, validated [45] and translated comprehensively into six European languages. These tools have been included in the first international RCT azithromycin study [29]. The measures are also being included in an international PCD registry developed as part of the BESTCILIA FP7 study, providing an international platform to systematically collect data on incidence, clinical presentation, treatment, and disease course. Qualitative studies that reflect different ethnicities and cultures are important and necessary to establish the needs and opinions specific to these groups.

## Conclusion

The findings of this review indicate the physical impact, emotional and stigmatising impact of PCD. They highlight the need for well-designed, quantitative studies using PCD-specific health-related quality of life measures to accurately determine the factors that impact PCD. There is also a need for the experience of patients to be further examined across ethnicities to evaluate various nuances between cultures. This will lead to better care, management, and outcomes for PCD patients.
